# Diabetes distress among immigrants of south Asian descent living in New York City: baseline results from the DREAM randomized control trial

**DOI:** 10.1186/s12889-025-21535-8

**Published:** 2025-02-02

**Authors:** Farhan Mohsin, Laura Wyatt, Hayley Belli, Shahmir Ali, Deborah Onakomaiya, Supriya Misra, Yousra Yusuf, Shinu Mammen, Jennifer Zanowiak, Sarah Hussain, Haroon Zafar, Sahnah Lim, Nadia Islam, Naheed Ahmed

**Affiliations:** 1 Institute for Excellence in Health Equity, NYU Grossman School of Medicine, 180 Madison Ave, New York, NY 10016 USA; 2 Department of Population Health, NYU Grossman School of Medicine, 180 Madison Ave, New York, NY 10016 USA; 3 Vilcek Institute of Graduate Biomedical Sciences, NYU Grossman School of Medicine, 180 Madison Ave, New York, NY 10016 USA; 4 Department of Public Health, San Francisco State University, 1600 Holloway Ave, San Francisco, CA 94132 USA

**Keywords:** Individuals of south Asian descent, Immigrants, Type 2 diabetes, Diabetes distress, Mental health, Immigrant health, Health disparities

## Abstract

**Background:**

Type 2 diabetes (T2D) disproportionately affects individuals of South Asian descent. Additionally, diabetes distress (DD) may lead to complications with diabetes management. This study examines the prevalence of DD among foreign-born individuals of South Asian descent in New York City (NYC) and its association with sociodemographic and clinical factors.

**Methods:**

Baseline data was collected from the Diabetes Research, Education, and Action for Minorities (DREAM) Initiative, an intervention designed to reduce hemoglobin A1c (HbA1c) among South Asian individuals with uncontrolled T2D at primary care practices in NYC. The Diabetes Distress Scale (DDS) measured DD, and Core Healthy Days Measures assessed physical and mental healthy days. Sociodemographic variables were analyzed using descriptive statistics, Chi-square tests assessed categorical variables, and Wilcoxon Rank Sum tests evaluated continuous variables (Type I error rate = 0.05). Logistic regression models examined associations between HbA1c, mental health, and other covariates with dichotomized DD subscales.

**Results:**

Overall, 414 participants completed the DDS at baseline (median age = 55.2 years; SD = 9.8). All were born outside of the US; the majority were born in Bangladesh (69.8%) followed by India, Pakistan, and Nepal (24.7%) and Guyana and Trinidad and Tobago (5.5%). High emotional burden, regimen-related distress and physician-related distress were reported by 25.9%, 21.9%, and 6.2% of participants, respectively. In adjusted analyses, individuals with ≥ 1 day of poor mental health had higher odds of overall distress (OR:3.8, *p* = 0.013), emotional burden (OR:4.5, *p* < 0.001), and physician-related distress (OR:4.6, *p* = 0.007) compared to individuals with no days of poor mental health. Higher HbA1c (OR:1.45, p = < 0.001) was associated with regimen-related distress; and lower emotional support was associated with overall distress (OR:0.92, *p* < 0.001) and regimen-related distress (OR:0.95, *p* = 0.012). Individuals born in Bangladesh had significantly lower odds of overall distress, emotional burden, and regimen-related distress compared to individuals born in Guyana and Trinidad and Tobago.

**Conclusions:**

Findings highlight the rate and risk factors of DD among individuals of South Asian descent living in NYC. Screening for DD in patients with prediabetes or diabetes should be integrated to address mental and physical health needs. Future research can benefit from a longitudinal analysis of the impact of DD on diabetes self-management and health outcomes.

**Trial registration:**

This study uses baseline data from “Diabetes Management Intervention for South Asians” (NCT03333044), which was registered with clinicaltrials.gov on 6/11/2017.

**Supplementary Information:**

The online version contains supplementary material available at 10.1186/s12889-025-21535-8.

## Background

Type 2 diabetes (T2D) is a leading cause of morbidity and mortality in the United States (US) and disproportionately affects individuals of South Asian descent [1–3], one of the fastest-growing ethnic minorities in the US and New York City (NYC) [4, 5]. Nationally, the 2011–2016 National Health and Nutrition Examination Survey revealed that individuals of South Asian descent had the highest prevalence of age- and sex-adjusted total diabetes (self-reported diabetes or undiagnosed diabetes using Hemoglobin A1c [HbA1c]) (23.3%) when compared to non-Hispanic White (12.1%) and non-Hispanic Asian individuals overall (19.1%) [6]. In NYC, the 2009–2012 Racial and Ethnic Approaches to Community Health Risk Factor Survey found the age-adjusted prevalence of self-reported diabetes to be highest among Asian Indians (19.0%) compared to Hispanic (16.5%), Black (14.3%), Korean (10.8%), and Chinese (9.3%) groups [7]; and data from the 2014–2018 NYC Community Health Survey found that Asian Indian individuals (21%) and underrepresented individuals of South Asian descent who identified as Bangladeshi, Bengali, Bhutanese, Nepali, Pakistani, and/or Sri Lankan (15%) had the highest prevalence of self-reported diabetes compared to NYC overall (11%) and Asian and Pacific Islander overall (12%) [8].

Living with T2D can be challenging due to the unique psychological impact the illness can have on an individual’s mental health [9]. Distress associated with diabetes may lead to complications and challenges with the management of diabetes [10–12], which may ultimately worsen diabetes-related outcomes. Diabetes distress (DD) is defined as the distinct emotional burdens or worries an individual may experience while trying to manage and live with diabetes [13]. Symptoms of DD may include feeling discouraged, worried, frustrated or tired of dealing with diabetes care or feeling as if diabetes is controlling oneself instead of the other way around [14].

The CDC reports that at any given 18-month period, 33–50% of people with diabetes have diabetes distress [14]. High levels of DD have been linked to low diabetes self-efficacy, poor glycemic control, and poor quality of life [15]. High DD levels are also associated with poor diet, high blood pressure (BP), lower medication adherence, and low physical activity [16]. Among individuals living in low- and middle-income South Asian countries, a scoping review identified a high prevalence of DD (18-76.2%), where high levels of DD were associated with lower medication adherence [17]. Cross-sectional studies from South Asian countries, such as Bangladesh (52.5%) and Pakistan (76.2%) reported high levels of DD, with the study in Pakistan identified lower education and income to be associated with DD [15, 18]. Mental illnesses, such as depression, have been linked with DD [18, 19]; nearly a third of participants in the Bangladesh study were found to have both DD and depressive symptoms [18]. A cross-sectional study among individuals of South Asian descent in Canada found a high prevalence of DD (52.5%), with total DD having a moderately positive correlation with depressive symptoms (*r* = 0.70, *p* < 0.001) [19].

Much of the existing literature on DD among South Asian subgroups has come from studies based in Canada and South Asian countries. To our knowledge, this is one of the first studies focusing on DD among individuals of South Asian descent with T2D living in the US. Combined with the rise of pre-diabetes in the US [20] and the high rates of DD elsewhere [15, 17, 18, 21], it is imperative to examine the impact of DD on individuals of South Asian descent living in the US. This study aims to examine the prevalence of DD among immigrants of South Asian descent in NYC and the association with socio-demographic characteristics and clinical measures [22, 23].

## Methods

### Study population

This study utilized baseline data from the randomized treatment group of the Diabetes Research, Education, and Action for Minorities (DREAM) Initiative, a multilevel diabetes management intervention designed to improve HbA1c among individuals of self-reported South Asian descent, including individuals of South Asian descent born in Bangladesh, Pakistan, India, Guyana, Nepal, and Trinidad and Tobago with uncontrolled T2D who were receiving care at small, community-based primary care practices in NYC serving largely South Asian populations [24]. Study recruitment protocols have been previously described [24]; briefly, individuals with: (1) HbA1c levels ≥7.0% in the past 12 months; (2) a visit to the provider’s office in the last 12 months; (3) between 21 and 74 years old; and (4) not pregnant at the time of screening were considered eligible to participate [24]. Lists of eligible participants were generated from electronic health record (EHR) patient lists at participating practices. Patients randomized into the treatment group were contacted by community health workers (CHWs) and invited to participate in the study. The study took place over three intervention waves. Each wave took place over six months and consisted of five monthly, CHW-led sessions lasting approximately 90–120 min. Baseline data were collected between July 2019 and August 2022. A total of 419 individuals were enrolled into the treatment group, and 414 of these individuals completed at least one subscale on the Diabetes Distress Scale (DDS) questionnaire at baseline and were included in this analysis. Written informed consent was obtained from all treatment group participants. The NYU Langone Health IRB ethics committee approved the study on November 28, 2017. Further details are described in the study protocol [24].

### Measures

Participant data was collected via electronic health records or from the screening and intake surveys. The intake survey was translated from English into Bengali/Bangla by staff.

#### Diabetes Distress

DD was measured using the DDS [13]. The DDS has been shown to have good internal reliability and validity and can serve as a tool to measure DD for research purposes to examine relationships between DD and blood glucose levels, diet, physical activity, and self-efficacy in the management of diabetes (Cronbach’s alpha = 0.93, with subscale ranging from 0.88 to 0.90) [16, 25]. Use of the DDS among South Asian subgroups, including Bangladeshi individuals, has found the scale to be a reliable tool to assess distress among Bangladeshi individuals living with T2D (Cronbach’s alpha = 0.84, with subscales ranging from 0.70 to 0.88 and the lowest score seen in physician-related distress) [9].

The original DDS consists of four subscales and 17 questions total: Emotional Burden (5 questions), Physician-related Distress (4 questions), Regimen-related Distress (5 questions), and Interpersonal Distress (3 questions). After the second wave of baseline data collection, CHWs reported that the questionnaire was long and burdensome to complete for participants. The questions were assessed by the study statistician, and one scale (Interpersonal Distress - Cronbach’s alpha = 0.882) was removed, as well as two questions from the Emotional Burden subscale (3 questions remained) and one question from Regimen-related Distress subscale (4 questions remained). No questions were removed from the Physician-related distress subscale (4 questions), with 11 questions total. Interpersonal distress was removed due to no difference in the subscale at baseline and follow-up for the first study wave. The Cronbach’s alpha for two waves of data collection with all questions was 0.940, while the Cronbach’s alpha for the reduced scale over three waves of data collection was 0.918. Cronbach’s alphas for each final subscale were: Regimen-related Distress: 0.894; Emotional Burden subscale: 0.916; and Physician-related distress: 0.821. See Supplementary File 1.

Respondents were asked to “Consider the degrees to which each item may have distressed or bothered you during the past month.” Responses for the DDS questions included: Not a problem [1], slight problem [2], moderate problem [3], Somewhat serious problem [4], serious problem [5], and very serious problem [6]. The mean of the questions was calculated for each subscale and all questions (the overall scale) ranged from 1 to 6. Informed by past research where a mean score of ≥ 3 was the threshold for being distressed, a dichotomous outcome was calculated for each subscale: low DD: <3 and high DD: ≥3 [26, 27].

#### Healthy days

Two questions from the CDC’s Core Healthy Days Measures were used to measure healthy days: “Now thinking about your physical health, which includes physical illness and injury, for how many days during the past 30 days was your physical health not good?” and “Now thinking about your mental health, which includes stress, depression, and problems with emotions, for how many days during the past 30 days was your mental health not good?” with responses ranging from 0 to 30 [28]. Each question was dichotomized into no days (0) and ≥ 1 day [1–30].

#### Emotional support

The 4-item PROMIS Emotional Support (4a) was used to assess perceived feelings of being cared for and valued and having confident relationships [29]. The raw total score ranges from 4 to 20; a t-score was calculated, ranging from 25.7 to 62.2, with a higher score representing higher levels of support [29].

#### Clinical measures

BP, weight, body mass index (BMI), and HbA1c were obtained from EHRs at each primary care site. Weight, BMI, and HbA1c were analyzed as continuous variables, and one categorical threshold was constructed for BP control: <130/80 [30–33].

#### Socio-demographics

Socio-demographic variables included sex (male or female), age (continuous), country of birth (choices included Bangladesh, Guyana, India, Nepal, Pakistan, Trinidad and Tobago, which were grouped into [1] Bangladesh [2], other South Asian country [Pakistan, India, and Nepal], and [3] Indo-Caribbean country [Guyana or Trinidad and Tobago]), years living in the US (continuous), marital status (married vs. not married), level of education (< high school, high school/some college, and college graduate or higher), and English spoken fluency (very well/well vs. not well/not at all). Age and sex were obtained from the EHR records used for eligibility. All other measures were obtained from the baseline surveys collected by the CHWs.

### Statistical analysis

Data were analyzed using SPSS version 28.0. Descriptive statistics were analyzed at baseline and stratified by sex and country of birth. Differences in sex and country of birth were assessed using Pearson Chi-square tests for categorical variables and Wilcoxon Rank Sum tests or ANOVAs for continuous variables. A type I error rate threshold of 0.05 was used to assess significance with no correction for multiple comparisons as all analyses were exploratory. Logistic regression was then performed to determine if HbA1c and mental health, along with other covariates, were associated with DD subscales first in unadjusted, univariable logistic regression. Variables found to be significant at *p* < 0.05 in the unadjusted, univariable regression were considered, and final adjusted models were fit to include variables significant at a type I error rate threshold of 0.05. Backwards stepwise selection was used to eliminate non-significant covariates in the final model. All final adjusted models included age, sex, education, country of birth, and years in the US, regardless of significance. Odds ratios (ORs), 95% confidence intervals (CIs), and p-values were calculated.

## Results

Table 1 presents socio-demographic, scale variables, and clinical measures overall and stratified by sex (*N* = 414). Median age was 55.2 years (SD = 9.8) and the majority of participants were married (93.2%). All individuals were born outside of the US. While the study focus was on individuals of South Asian descent in NYC, the study sample was primarily composed of individuals born in Bangladesh (69.8%), followed by India (14.5%), Pakistan (8.0%), Guyana (4.3%), Nepal (2.2%), and Trinidad and Tobago (1.2%). Over a quarter (25.9%) reported high emotional burden, 6.2% high physician-related distress, and 21.9% high regimen-related distress.

**Table 1:** Baseline characteristics of the sample, overall and stratified by sex (*n* = 414)

	Overall (*n* = 414)		Female (*n* = 221)		Male (*n* = 193)		
	n	%	n	%	n	%	p-value
Age							
Mean (SD)	55.2 (9.8)		53.9 (9.4)		56.6 (10.0)		0.006
Education							< 0.001
Less than high school	180	43.7	126	57.5	54	28.0	
High school/ Some college	135	32.8	61	27.9	74	38.3	
College graduate or higher	97	23.5	32	14.6	65	33.7	
Marital status							< 0.001
Married	384	93.2	194	88.6	190	98.4	
Divorced/Widowed/Separated	28	6.8	25	11.4	3	1.6	
English fluency							< 0.001
Very well/Well	163	39.7	59	27.1	104	53.9	
Not well/Not at all	248	60.3	159	72.9	89	46.1	
Country of birth							0.478^a^
Bangladesh	289	69.8	153	69.2	136	70.4	
India	60	14.5	31	14.0	29	15.0	
Pakistan	33	8.0	22	10.0	11	5.7	
Nepal	9	2.2	0	0.0	17	4.7	
Guyana	18	4.3	12	5.4	6	3.1	
Trinidad and Tobago	5	1.2	3	1.4	2	1.0	
Years lived in the US							
Mean (SD)	14.2 (9.1)		12.4 (7.7)		16.2 (10.0)		< 0.001
Mental Health Days							0.008
No days	336	85.9	167	81.5	169	90.9	
≥1 days	55	14.1	38	18.5	17	9.1	
Physical Health Days							0.004
No days	326	83.0	161	77.8	165	88.7	
≥1 days	67	17.0	46	22.2	21	11.3	
Emotional Support t-score							
Mean (SD)	53.8 (8.9)		52.2 (9.3)		55.7 (8.1)		< 0.001
HbA1c							
Median (IQR)	8.4 (1.4)		8.5 (1.4)		8.4 (1.3)		0.510
Weight							
Mean (SD)	159.9 (28.1)		151.8 (26.8)		169.2 (26.6)		< 0.001
BMI							
Mean (SD)	27.8 (4.2)		28.3 (4.4)		27.1 (3.8)		0.004
BP Control (< 130/80)							0.868
Controlled	166	40.2	88	39.8	78	40.6	
Uncontrolled	247	59.8	133	60.2	114	59.4	
**Diabetes Distress Scales**							
DDS Total, Continuous							
Mean (SD)	2.0 (1.0)		2.1 (1.0)		1.8 (0.9)		< 0.001
DDS Total, Categories							0.031
Low (< 3)	332	84.1	167	80.3	165	88.2	
High (≥ 3)	63	15.9	41	19.7	22	11.8	
Emotional Burden, Continuous							
Mean (SD)	2.3 (1.4)		2.5 (1.5)		2.1 (1.3)		0.001
Emotional Burden, Categories							0.058
Low (< 3)	304	74.1	154	70.3	150	78.5	
High (≥ 3)	106	25.9	65	29.7	41	21.5	
Physician-Related Distress, Continuous							
Mean (SD)	1.5 (0.8)		1.5 (0.8)		1.4 (0.7)		0.074
Physician-Related Distress, Categories							0.459
Low (< 3)	380	93.8	199	92.6	181	94.8	
High (≥ 3)	25	6.2	16	7.4	10	5.2	
Regimen-Related Distress, Continuous							
Mean (SD)	2.2 (1.3)		2.4 (1.3)		1.9 (1.2)		< 0.001
Regimen-Related Distress, Categories							0.011
Low (< 3)	318	78.1	159	71.9	159	83.7	
High (≥ 3)	89	21.9	58	26.2	31	16.3	

DDS, Diabetes Distress Scale; BP, Blood pressure; SD, standard deviation; US, United States

^a^p-value for Bangladesh vs. India, Pakistan, Nepal vs. Trinidad and Tobago or Guyana

When stratifying by sex, males were significantly more likely than females to have a college degree or higher (33.7% vs. 14.6%), to be married (98.4% vs. 88.6%), to speak English very well or well (53.9% vs. 27.1%), and to have lived in the US for longer. Females were significantly more likely to report having ≥ 1 day of poor mental or physical health (18.5% vs. 9.1% and 22.2% vs. 11.3%, respectively), and to have a higher mean emotional support t-score. Females had a significantly higher mean score for the emotional burden and regimen-related distress subscales; when dichotomized, high regimen-related stress was significantly greater among females compared to males (26.2% vs. 16.3%, *p* = 0.011).

When stratifying by country of birth, significant differences were seen by age, education, marital status, English fluency, years lived in the US, mental and physical health days, emotional support, HbA1c, weight, BMI, and all DDS measures. Individuals born in Guyana or Trinidad and Tobago had the highest DD scores, followed by individuals born in India, Pakistan, or Nepal (Table 2).

**Table 2:** Baseline characteristics of the sample, overall and stratified by COB (*n* = 414)

	Bangladesh		India, Pakistan, Nepal		Guyana or Trinidad and Tobago		
	n	%	n	%	n	%	p-value
Sex							0.478
Female	154	52.9	54	51.4	15	65.2	
Male	137	47.1	51	48.6	8	34.8	
Age							
Mean (SD)	54.5 (10.1)		56.9 (8.7)		54.9 (9.0)		0.002
Education							< 0.001
Less than high school	82	28.3	85	81.0	14	63.6	
High school/ Some college	116	40.0	16	15.2	7	31.8	
College graduate or higher	92	31.7	4	3.8	1	4.5	
Marital status							< 0.001
Married	270	93.1	103	99.0	16	69.6	
Divorced/Widowed/Separated	20	6.9	1	1.0	7	30.4	
English fluency							< 0.001
Very well/Well	129	44.8	18	17.1	18	78.3	
Not well/Not at all	159	55.2	87	82.9	5	21.7	
Years lived in the US							
Mean (SD)	13.0 (9.2)		17.1 (7.9)		16.2 (9.5)		< 0.001
Mental Health Days							0.004
No days	229	83.3	96	95.0	14	73.7	
≥1 day	46	16.7	5	5.0	5	26.3	
Physical Health Days							< 0.001
No days	220	79.4	99	97.1	11	57.9	
≥1 day	57	20.6	3	2.9	8	42.1	
Emotional Support t-score							
Mean (SD)	56.1 (8.2)		48.2 (8.5)		50.2 (8.0)		< 0.001
HbA1c							
Mean (SD)	8.3 (1.3)		8.6 (1.5)		9.0 (1.8)		0.042
Weight							
Mean (SD)	155.7 (24.8)		171.6 (30.4)		161.5 (37.3)		< 0.001
BMI							
Mean (SD)	27.3 (3.6)		28.9 (5.1)		28.0 (4.9)		< 0.001
BP Control (< 130/80)							0.158
Controlled	123	42.3	35	33.7	12	52.2	
Uncontrolled	168	57.7	69	66.3	11	47.8	
**Diabetes Distress Scales**							
DDS Total, Continuous							
Mean (SD)	1.6 (0.6)		2.8 (1.0)		3.0 (1.4)		< 0.001
DDS Total, Categories							< 0.001
Low (< 3)	262	96.0	56	56.6	14	60.9	
High (≥ 3)	11	4.0	43	43.4	9	39.1	
Emotional Burden, Continuous							
Mean (SD)	1.7 (0.9)		3.6 (1.6)		3.8 (1.7)		< 0.001
Emotional Burden, Categories							< 0.001
Low (< 3)	259	90.6	38	37.6	7	30.4	
High (≥ 3)	27	9.4	63	62.4	16	69.6	
Physician-Related Distress, Continuous							
Mean (SD)	1.3 (0.6)		1.8 (0.9)		2.1 (1.4)		< 0.001
Physician-Related Distress, Categories							< 0.001
Low (< 3)	270	96.4	92	90.2	18	78.3	
High (≥ 3)	10	3.6	10	9.8	5	21.7	
Regimen-Related Distress, Continuous							
Mean (SD)	1.7 (0.8)		3.2 (1.5)		3.3 (1.4)		< 0.001
Regimen-Related Distress, Categories							< 0.001
Low (< 3)	259	91.2	49	49.0	10	43.5	
High (≥ 3)	25	8.8	51	51.0	13	56.5	

DDS, Diabetes Distress Scale; BP, Blood pressure; SD, standard deviation; US, United States

Univariate regression analyses found differences in dichotomized DD subscales (*p* < 0.05) for the following variables: DDS overall (sex, age, education, English fluency, country of birth, mental health days, emotional support t-score, and HbA1c); emotional burden subscale (age, education, English fluency, country of birth, mental health days, emotional support t-score, BMI, and HbA1c); physician-related distress subscale (country of birth, mental health days, and emotional support t-score); and regimen-related distress (sex, education, English fluency, country of birth, emotional support t-score, BMI, weight, HbA1c) (Table 3). These variables were then each tested for inclusion in a final adjusted regression model, as applicable for the outcome.

**Table 3:** Unadjusted Univariate regression models predicting high risk DD subscales

	DDS - overall ≥ 3			Emotional burden ≥3			Physician-related distress ≥ 3			Regimen-related distress ≥ 3		
	OR	95% CI	p-value	OR	95% CI	p-value	OR	95% CI	p-value	OR	95% CI	p-value
**Sex (Ref = Male)**												
Female	1.8	1.1, 3.2	**0.033**	1.5	1.0, 2.4	0.059	1.4	0.6, 3.1	0.461	1.9	1.1, 3.0	**0.012**
**Age**,** continuous**	1.03	1.00, 1.06	**0.035**	1.04	1.02, 1.07	**0.001**	1.02	0.98, 1.06	0.383	1.02	1.00, 1.05	0.125
**Education (Ref = College graduate or higher)**												
Less than high school	19.9	4.7, 84.0	**< 0.001**	15.5	6.0, 40.1	**< 0.001**	2.6	0.9, 8.0	0.090	9.3	3.8, 22.4	**< 0.001**
High school/ Some college	3.1	0.6, 14.9	**0.160**	2.6	0.9, 7.4	**0.065**	0.5	0.1, 2.5	0.426	1.8	0.7, 4.9	0.249
**Marital Status (Ref = Not married)**												
Married	4.6	0.6, 34.4	0.141	1.2	0.5, 3.1	0.687	1.8	0.2, 13.6	0.582	2.0	0.7, 7.6	0.204
**English Fluency (Ref = Very well/ well)**												
Not well/Not at all	3.7	1.9, 7.3	**< 0.001**	2.6	1.6, 4.3	**< 0.001**	1.5	0.6, 3.5	0.399	2.3	1.4, 4.0	**0.002**
**Country of birth (Ref = Guyana or Trinidad and Tobago)**												
India, Pakistan, Nepal	1.2	0.5, 3.1	0.707	0.7	0.3, 1.9	0.519	0.4	0.1, 1.3	0.391	0.8	0.3, 2.0	0.633
Bangladesh	0.1	0.0, 0.2	**< 0.001**	0.0	0.0, 0.1	**< 0.001**	0.1	0.0, 0.4	**< 0.001**	0.1	0.0, 0.2	**< 0.001**
**Years in the US**,** continuous**	1.0	0.99, 1.05	0.182	1.04	1.02, 1.07	0.183	0.99	0.94, 1.04	0.661	1.00	0.96, 1.03	0.875
**Mental Health Days (Ref = No days)**												
≥1 day	2.0	1.0, 4.1	**0.048**	1.8	1.0, 3.4	**0.049**	4.1	1.6, 10.2	**0.003**	1.50	0.8, 2.8	0.263
**Physical Health Days (Ref = No days)**												
≥1 day	0.8	0.4, 1.8	0.621	1.0	0.6, 1.9	0.884	1.7	0.7, 4.5	0.275	1.2	0.6, 2.3	0.626
**Emotional support t-score**,** continuous**	0.88	0.85, 0.92	**< 0.001**	0.92	0.89, 0.94	**< 0.001**	0.96	0.92, 1.00	0.063	0.91	0.88, 0.93	**< 0.001**
**BMI**,** continuous**	1.03	0.96, 1.09	0.112	1.06	1.00, 1.12	**0.028**	0.98	0.89, 1.09	0.703	1.07	1.02, 1.13	**0.011**
**Weight**,** continuous**	1.00	0.99, 1.01	0.445	1.01	1.00, 1.02	0.067	1.00	0.98, 1.01	0.882	1.01	1.00, 1.02	**0.035**
**HbA1c**,** continuous**	1.31	1.10, 1.56	**0.003**	1.20	1.03, 1.40	**0.019**	1.12	0.92, 1.54	0.188	1.38	1.18, 1.62	**< 0.001**
**BP Control (Ref = < 130/80)**												
≥130/80	1.3	0.7, 2.3	0.384	1.3	0.9, 2.1	0.206	0.7	0.3, 1.6	0.407	1.3	0.8, 2.0	0.362

DDS, Diabetes Distress Scale; BP, Blood pressure; SD, standard deviation; US, United States

**Table 4:** Adjusted regression models predicting high risk DD subscales

	DDS - overall ≥ 3			Emotional burden ≥3			Physician-related distress ≥ 3			Regimen-related distress ≥ 3		
	OR	95% CI	p-value	OR	95% CI	p-value	OR	95% CI	p-value	OR	95% CI	p-value
**Sex (Ref = Male)**												
Female	1.0	0.4, 2.2	0.974	1.3	0.7, 2.6	0.424	1.0	0.4, 2.8	1.000	1.2	0.6, 2.3	0.634
**Age**,** continuous**	1.01	0.97, 1.06	0.662	1.03	0.99, 1.07	0.070	1.02	0.96, 1.07	0.573	1.00	0.96, 1.03	0.816
**Education (Ref = College graduate or higher)**												
Less than high school	3.8	0.7, 21.0,	0.120	4.4	1.5, 13.3	**0.009**	1.4	0.4, 5.7	0.620	3.5	1.2, 10.7	0.026
High school/ Some college	3.1	0.6, 17.8	0.195	2.3	0.8, 7.2	0.140	0.5	0.1, 2.7	0.454	1.9	0.6, 6.0	0.248
**Country of birth (Ref = Guyana or Trinidad and Tobago)**												
India, Pakistan, Nepal	1.2	0.3, 4.3	0.757	0.6	0.2, 1.9	0.354	0.5	0.1, 2.5	0.425	1.0	0.3, 3.2	0.989
Bangladesh	0.1	0.0, 0.4	**0.002**	0.1	0.0, 0.2	**< 0.001**	0.2	0.0, 1.0	0.057	0.2	0.1, 0.6	**0.006**
**Years in US**,** continuous**	0.99	0.94, 1.03	0.509	1.02	0.98, 1.06	0.302	0.99	0.93, 1.04	0.660	1.00	0.96, 1.03	0.771
**Mental Health Days (Ref = No days)**												
≥1 day	3.8	1.3, 11.1	**0.013**	4.5	1.8, 10.8	**< 0.001**	4.6	1.5, 14.0	**0.007**	1.8	0.8, 4.3	0.187
**Emotional support t-score**	0.92	0.88, 0.97	**< 0.001**	0.99	0.95, 1.02	0.430	1.02	0.96, 1.08	0.572	0.95	0.92, 0.99	**0.012**
**HbA1c**,** continuous**	1.27	1.00, 1.60	0.050	1.15	0.92, 1.43	0.216	1.21	0.90, 1.63	0.214	1.45	1.17, 1.79	**< 0.001**

DDS, Diabetes Distress Scale; US, United States

Models are adjusted for all variables presented in the table

After conducting adjusted regression analyses (Table 4), we found the following results:

### DDS - overall

When adjusting for other variables in the model, individuals born in Bangladesh (OR = 0.1, 95% CI: 0.0, 0.4, *p* < 0.001) had significantly lower odds of overall distress compared to individuals born in Guyana or Trinidad and Tobago; individuals with ≥ 1 day of poor mental health in the past month had higher odds of overall distress (OR = 3.8, 95% CI: 1.3, 11.1 *p* = 0.013) compared to individuals with no days of poor mental health; and lower emotional support was significantly associated with overall distress (OR = 0.92, 95% CI: 0.88, 0.97, *p* < 0.001).

### DDS - emotional burden

When adjusting for other variables in the model, individuals born in Bangladesh (OR = 0.1, 95% CI: 0.0, 0.2, *p* < 0.001) had significantly lower odds of *DDS emotional burden* compared to individuals born in Guyana or Trinidad and Tobago; individuals with ≥ 1 day of poor mental health in the past month had significantly higher odds of *DDS emotional burden* (OR = 4.5, 95% CI: 1.8, 10.8, *p* < 0.001) compared to individuals with no days of poor mental health; and individuals with less than high school education had significantly higher odds of *DDS emotional burden* (OR = 4.4, 95% CI: 1.5, 13.3, *p* = 0.009) compared to individuals with a college degree or higher.

### DDS - physician-related distress

When adjusting for other variables in the model, individuals with ≥ 1 day of poor mental health in the past month had significantly higher odds of *DDS physician-related distress* (OR = 4.6, 95% CI: 1.5, 14.0, *p* = 0.007) compared to individuals with no days of poor mental health.

### DDS - regimen-related distress

When adjusting for other variables in the model, individuals born in Bangladesh (OR = 0.2, 95% CI: 0.1, 0.6, *p* = 0.006) had significantly lower odds of *DDS regimen-related distress* compared to individuals born in Guyana or Trinidad and Tobago; lower emotional support was significantly associated with *DDS regimen-related distress* (OR = 0.95, 95% CI: 0.92, 0.99, *p* = 0.012); and higher HbA1c was significantly associated with *DDS regimen-related distress* (OR = 1.45, 95% CI: 1.17, 1.79, *p* < 0.001).

## Discussion

In our sample of individuals of South Asian descent living with T2D in NYC, 15.9% reported having high overall diabetes distress. This is notably lower than previous studies in Bangladesh, Canada, China, and Malaysia, where the prevalence of DD ranged from 18.0 to 76.2% [17, 34–37]. Meta-analyses of the prevalence of DD in individuals with T2D in the US also reveal a wide range of prevalence of DD (19-79.5%) [38]. Findings from these studies suggest that the prevalence of DD may vary across and within countries and healthcare settings. The discrepancy between the DD prevalence in previous studies compared to this study may result from methodological differences in the study design, data collection, or analysis methods. Additionally, our sample likely benefitted from higher-quality diabetes care, as study participation required at least one primary care visit in the past year, which may explain the lower observed prevalence of DD in this study compared to other studies. These discrepancies underscore how factors such as socioeconomic status, education levels, healthcare access and available healthcare resources influence the prevalence and management of DD.

A significantly greater proportion of females (19.7%) in our sample had high DD when compared to males (11.8%). Previous studies have reported that there may be a link between persistence of DD, which is defined as the occurrence of DD across multiple study time points, and being female [38, 39]. This increased persistence of DD among females with T2D may be attributable to sex-based differences: psychological stress among females is higher compared to males [40], with females encountering more stressful life events and being more negatively impacted by stressful life events when compared to males [41, 42]. Societal expectations may also influence the reporting of DD among males, who may be less likely to report distress due to cultural norms that may discourage men from expressing vulnerability or sharing emotions of distress [43]. Further research on developing effective interventions that address the unique gender-specific dynamics and challenges faced by female and male in managing T2D and DD is needed.

In adjusted analyses, individuals of South Asian descent born in Bangladesh had significantly lower odds of overall DD as well as significantly lower odds of emotional burden and regimen-related distress compared to individuals of South Asian descent born in Indo-Caribbean countries (Guyana or Trinidad and Tobago). Evidence indicates that a network of community- or family-level factors may improve the health outcomes via social support [44]. Social support has been reported to serve as a protective factor for DD [45], and evidence indicates that Bangladeshi immigrant households tend to be “family-based” (multigenerational families living together) [46]. This suggests that the participants born in Bangladesh from this sample may have additional sources of social support, which may confer them to be more resilient to distress when compared to the Indo-Caribbeans in the sample. The unique context of ethnic enclaves in NYC further complicates the understanding of health outcomes in these populations [47]. The Bangladeshi community in NYC has experienced significant growth in recent decades [48], which may potentially foster stronger social networks and access to culturally relevant resources. However, these may not be uniformly experienced across all ethnic groups [49]. Additionally, systemic barriers may exacerbate the risk for DD for individuals of South descent born in Indo-Caribbean countries. Understanding these disparities is crucial for developing tailored interventions that address the unique challenges faced by these communities.

Higher emotional support significantly reduces the odds of overall DD and regimen-related distress. Having confident relationships and perceived feelings of being cared for by one’s family and friends can affect one’s ability to manage T2D [50]. One study found that providing emotional support via phone calls or text messages prompted better self-management of T2D for individuals living with T2D [51]. Another study showed that enhancing emotional support can be a useful strategy to help reduce the burden of diabetes distress and encourage better self-management of diabetes [52].

Adjusted analyses also show that elevated HbA1c levels are associated with higher odds of regimen-related distress, while participants with ≥ 1 day of poor mental health had increased odds of overall, emotional burden, and physician-related distress. A San Francisco study among white and non-white adult T2D patients found increases in distress was associated with poorer HbA1c outcomes [16]. A scoping review also indicated a significant association between HbA1c levels and DD among individuals of South Asians descent in low- and middle-income countries [17]. These findings align with our study as they also highlight the bidirectional relationship between glycemic control and diabetes distress. Additionally, participants with ≥ 1 day of poor mental health may have existing mental health challenges such as depression or anxiety, making them more vulnerable to DD. Several studies have shown a significant correlation between DD and depression and anxiety [27, 37, 53], explaining why those with ≥ 1 day of poor mental health may have increased odds of DD. Our findings reveal that identifying and treating these risk factors may reduce the burden of DD among individuals of South Asian descent living with T2D; in addition, these groups may be particularly important to include in diabetes management efforts.

Lower education attainment was significantly associated with higher emotional burden compared having a college education. Education is an important social determinant of health, where low attainment has been linked to health inequities [54]. Studies in Pakistan and Saudi Arabia reported similar findings related to education; a higher emotional burden was found to be significantly associated with low education [15, 55]. Low education is also often associated with decreased health literacy, poorer health outcomes and poorer adherence to health behaviors [56, 57], all of which may impair one’s ability to manage diabetes, and contribute to higher emotional burden and regimen-related distress.

Data from the 2021 American Community Survey in NYC found that adults who have not graduated from high school had a higher proportion of uninsured individuals when compared to individuals with a Bachelor’s degree or higher [58]. The lack of health insurance may weaken an individual’s ability to consult a physician or afford diabetes medication, leading to increased emotional burden. Qualitative studies have also found that insurance barriers and diabetes burnout contribute to distress and suboptimal health and self-management outcomes [59, 60]. While these studies did not focus on individuals of South Asian descent [59, 60], these issues are relevant for South Asian descendants in the US, who may face similar challenges with insurance coverage and access to affordable healthcare in addition to stressors such as acculturative stress, discrimination, and socioeconomic hardships, which may further complicate diabetes management.

### Study Limitations

Although this is one of the first studies to examine the prevalence and predictors of DD among individuals of South Asian descent living in the US, some limitations must be acknowledged. First, measures were collected by CHWs, which may lead to response bias. Although CHWs received formal training to mitigate the possibility of response bias, participants may have felt the need to respond with positive answers. Second, this was a cross-sectional, observational study design, and therefore while covariate adjustment was included in models, causality of relationships is limited. Third, the DDS was modified for this study in order to reduce participant burden, resulting in a shortened version of the scale. This could affect the comparability of the study findings with other studies that used the full DDS or the subscales. Fourth, our Guyana or Trinidad and Tobago referent group was small (*n* = 23), which may reduce the generalizability. As our study was conducted at a setting with a predominantly large population of individuals born in Bangladesh, the study findings may not be generalizable to other minority communities. Future research should strive to sample a larger, more varied population of individuals of South Asian descent living in the US.

## Conclusions

This study highlights the low prevalence of DD among individuals of South Asian descent in NYC, with Bangladeshi participants having significantly lower odds of DD compared to those born in Guyana or Trinidad and Tobago. Associations with a higher odds of DD included being female, low educational attainment, elevated HbA1c levels, and those with have ≥ 1 poor mental health day per month. These findings contribute to the literature by highlighting the prevalence of DD among individuals of South Asian descent in NYC and factors associated with DD, underscoring DD as a medically relevant issue that impacts the patient ability to successfully manage T2D.

It is important to acknowledge that concept validation, which assesses the viability of survey tools or measures, for these diverse cultural groups are currently missing. Cultural validity when developing instruments can help to accurately capture the experiences of diverse populations [61]. Future research can benefit from establishing concept validation prior to scale validation to ensure that the measures employed, such as the DDS and related questionnaires, accurately reflect the experiences of the populations being studied. Interventional and health policy research should also screen for DD in prediabetes/diabetes patients and integrate mental and physical health services during medical appointments. Longitudinal studies on the impact of DD on diabetes self-management, and mental and physical health, would provide insight into causal relationships that may exist between DD with sociodemographic characteristics and clinical measures. Addressing the complex interplay between diabetes distress, mental health and diabetes management is essential for improving health outcomes, and by prioritizing culturally relevant validated measures and integrating care models that screen for DD, we can better identify at-risk populations and ensure more equitable and effective diabetes care.

## Electronic supplementary material

Below is the link to the electronic supplementary material.

Supplementary Material 1

Supplementary Material 2

## Data Availability

The data and materials generated during this study are not publicly available, as the study is ongoing, but will be available from the corresponding author upon reasonable request.

## Abbreviations

BP: Blood pressure
BMI: Body mass index
CHW: Community health worker
CI: Confidence interval
DD: Diabetes Distress
DDS: Diabetes Distress Scale
DREAM: Diabetes Research, Education, and Action for Minorities Initiative
EHR: Electronic health record
HbA1c: Hemoglobin A1c
NYC: New York City
OR: Odds ratio
T2D: Type 2 diabetes
