# Supplementary material for: Diabetes distress among immigrants of south Asian descent living in New York City: baseline results from the DREAM randomized control trial
Source: BMC Public Health. 2025 Feb 2;25:422. doi: 10.1186/s12889-025-21535-8 (PMC11789405; doi:10.1186/s12889-025-21535-8)
Supplement: Supplementary file 1 — Supplementary Material 1 [file 12889_2025_21535_MOESM1_ESM.docx]

Supplementary File 1. DDS Questions included in all three waves.

| DDS Questions | Included in all waves |
| --- | --- |
| **Emotional Burden** |  |
| Feeling that diabetes is taking up too much of my mental and physical energy every day | X |
| Feeling angry, scared, and/or depressed when I think about living with diabetes | X |
| Feeling that diabetes controls my life | X |
| Feeling that I will end up with serious long-term complications, no matter what I do |  |
| Feeling overwhelmed by the demands of living with diabetes |  |
| **Physician-related Distress** |  |
| Feeling that my doctor doesn’t know enough about diabetes and diabetes care | X |
| Feeling that my doctor doesn’t give me clear enough directions on how to manage my diabetes | X |
| Feeling that my doctor doesn’t take my concerns seriously enough | X |
| Feeling that I don’t have a doctor who I can see regularly enough about my diabetes | X |
| **Regimen-related Distress** |  |
| Feeling that I am not testing my blood sugars frequently enough |  |
| Feeling that I am often failing with my diabetes routine | X |
| Not feeling confident in my day-to-day ability to manage diabetes | X |
| Feeling that I am not sticking closely enough to a good meal plan | X |
| Not feeling motivated to keep up my diabetes management plan | X |
| **Interpersonal Distress** |  |
| Feeling that friends or family are not supportive enough of self-care efforts (e.g., planning activities that conflict with my schedule, encouraging me to eat the wrong foods) |  |
| Feeling that friends or family don’t appreciate how difficult living with diabetes can be |  |
| Feeling that friends or family don’t give me the emotional support that I would like |  |
