# Supplementary material for: Diabetes distress among immigrants of south Asian descent living in New York City: baseline results from the DREAM randomized control trial
Source: BMC Public Health. 2025 Feb 2;25:422. doi: 10.1186/s12889-025-21535-8 (PMC11789405; doi:10.1186/s12889-025-21535-8)
Supplement: Supplementary file 2 — Supplementary Material 2 [file 12889_2025_21535_MOESM2_ESM.pdf]

NYU School of Medicine
NYU LANGONE MEDICAL CENTER

Office of Science and Research
Institutional Review Board
1 Park Ave.| 6th Floor, | New York, NY 10016

# Approval of Submission November 27, 2017

Dear Nadia Islam:

On 11/27/2017 4:44 PM EST, the IRB reviewed the submission below: All conditions for approval were met on 11/27/2017.

| | |
|---|---|
| principal investigator | Nadia Islam |
| email | Nadia.islam@nyumc.org |
| study number | i17-01479 |
| study title | Integrated Community-Clinical Linkage Model to Manage Diabetes among South Asians |
| performance period | 11/27/2017 to 11/26/2018 inclusive. Before 11/26/2018 or within 30 days of study closure, whichever is earlier, you are to submit a continuing review with required explanations. You can submit a continuing review by navigating to the active study and clicking Create Modification / CR.<br>If continuing review approval is not granted before the expiration date of 11/26/2018, approval of this study expires on that date. |
| location(s) | Translational Research Building (NYUMC Locations), Private Practice (Other Locations) |
| sponsor(s) | Name: National Institutes of Health (NIH); Funding Source ID: U54MD000538-15, |
| review type | Initial Study [Expedited Categories 5, 6, 7] |
| board name | All Boards |
| materials approved for use | • 17-01479 Protocol IRB Edits 11-27-2017_clean.pdf, Category: IRB Protocol<br>• 17-01479 Consent_Brochure_CHW Intervention_English_IRB Edits 11-27-2017_clean.pdf, Category: Consent Form<br>• Consent Signature Page_CHW Intervention_English_10.9.pdf, Category: Consent Form<br>• Verbal_ Consent_Script_Key_Informant_Interview_11.1.17_s17-01479.pdf, Category: Consent Form<br>• Application for waiver of documentation of consent_Provider Survey and Key Informant Interview_11.1.17_s17-01479.pdf, Category: Consent Form<br>• app.waiver.of.authorization._Telephone Screening Form_s17-01479.pdf, Category: Consent Form<br>• Key_Informant_Interview Guide_s1701479.pdf, Category: IRB Protocol<br>• Patient Baseline Survey_s17-01479.pdf, Category: IRB Protocol<br>• 6_month_followup_Survey_s17-01479.pdf, Category: IRB Protocol<br>• Provider_Survey_U54_s1701479.pdf, Category: IRB Protocol<br>• Progress_Note_CHW Intervention_11.1_s1701479.pdf, Category: IRB Protocol<br>• Script_for_Telephone Screening_11.1.17_s1701479.pdf, Category: Recruitment Materials<br>• Telephone_Screening Form_11.1.17_s1701479.pdf, Category: Recruitment Materials<br>• Recruitment_Letter_CHW Intervention_s1701479_English_10.5.17.pdf, Category: Recruitment Materials<br>• Invitation and Elements of Informed Consent_Provider Survey_11.1.17_s17-01479.pdf, Category: Recruitment Materials<br>• Invitation and Elements of Informed Consent Text_Key Informant Interview_10.13.17.pdf, Category: Recruitment Materials<br>• U54 Full Center Grant Application, Category: Sponsor Attachment<br><br>A waiver of documentation of Informed Consent has been granted in accordance with 45 CFR 46.117 (c) (2); since the study presents minimal risk of harm to subjects and involves no procedures for which written consent in normally required outside the research. |
| #of subjects approved to consent | 4069 |

| | |
|---|---|
| vulnerable populations approved for participation in this study | |

The current IRB Status of your study is: Approved. This study was reviewed by the NYU School of Medicine's Institutional Review Board (IRB). During the review of your study, the IRB specifically considered:

1. the risks and anticipated benefits (if any) to your subjects
2. the selection of subjects
3. the procedures for securing and documenting informed consent
4. the safety of your subjects
5. the privacy of your subjects and confidentiality of the data

*Your study cannot commence until all ancillary review decisions are complete.  In order to determine the state of all ancillary reviews please go the My Studies page of this study in Research Navigator. Ancillary review statuses will be found on the right side of the header section.*

*Please note; if your study includes a clinical trial agreement or budget you will need to ensure approval has been issued from My Agreements/CRMS and The Office of Clinical Trials before you proceed with any aspects of this study including the enrollment of human subjects.*

**Review Notes**

For NIH Grant funded research: the IRB has found the IRB approved protocol referenced above to be consistent with the NIH grant application.

Helen Panageas (signature) November 27, 2017

RE: Study#i17-01479

Helen Panageas, Director, Institutional Review Board OHRP #FWA00004952

**Notes**

- You must submit all changes to this study (e.g., protocol, recruitment materials, consent forms, etc.) via eSubmission to the IRB for review and approval prior to initiation of the change(s), except where necessary to eliminate apparent immediate hazards to the subject(s). Changes made to eliminate apparent immediate hazards to subjects must be reported to the IRB within 24 hours.
- You must report all adverse and/or unanticipated event(s) that occur during the course of this study to IRB via eSubmission in accordance with IRB Policy.
- Use only IRB-approved copies of your consent form(s), questionnaire(s), letter(s), advertisement(s), etc. in your study. Do not use expired consent forms.
- You must inform all research staff listed on this study of changes or adverse events that occur.
- IRB's approval is valid until the end date of the performance period indicated above. A reminder for renewal should be e-mailed to you from the IRB 90, 60 and 30 days before this study's approval is scheduled to expire. However, you are responsible for submitting all renewal materials at least eight weeks before expiration regardless of whether or not you receive a reminder notice.
- All IRB policy documents can be found on our website: http://irb.med.nyu.edu/library
- Prior to initiating an IRB-approved study, you must receive written approval from an authorized representative for each site where your study will take place. Key contacts are:
  - **Bellevue Hospital**: when Bellevue Hospital is listed as a site where your study can take place, please note that you may have to complete additional work in BHC’s Reason system. Bellevue will be contacting you with any additional needed information. For questions on Bellevue Hospital research, please contact *BellevueResearch@bellevue.nychhc.org*

   - CTSI - Clinical and Translational Science Institute, NYU School of Medicine [formerly General Clinical Research Center (GCRC)], ctsi@nyumc.org.
   - NYU Langone Medical Center (Tisch Hospital/Rusk Institute/Co-op Care/HJD/Perlmutter Cancer Center) site approval is handled for you automatically (as needed) by the Office of Clinical Trials

- The IRB may terminate studies that are not in compliance with NYU Langone Medical Center/School of Medicine Policies & Procedures and the requirements of the Institution's Federal Wide Assurance with the Federal Government. Direct IRB questions, correspondence and forms (e.g., continuing reviews, amendments, adverse events, etc.) to 212-263-4110 or IRB-INFO@nyumc.org.
- Prior to initiating an IRB-approved study, you must receive written approval from an authorized representative of the Office of Clinical Trials. You may contact the Office of Clinical Trials at 212.263.4210 or clinicaltrials@nyumc.org.

*NYU SoM IRB operates in accordance with Good Clinical Practices (GCP) and applicable laws and regulations. The NYU SoM IRB Federal Wide Assurance number is 00004952.*
